# Real-World Study of US Adults with Paroxysmal Nocturnal Hemoglobinuria Treated with Pegcetacoplan

**DOI:** 10.3390/hematolrep16040065

**Published:** 2024-10-29

**Authors:** Brian Mulherin, Apeksha Shenoy, Lily Arnett, Weiqi Jiao, Joseph Guarinoni, Sujata Sarda, Jinny Min, David Dingli

**Affiliations:** 1 Hematology Oncology of Indiana, Indianapolis, IN 46260, USA; brian.mulherin@aoncology.com; 2 Ascension St. Vincent Indianapolis, Indianapolis, IN 46260, USA; 3 Boston Strategic Partners Inc., Boston, MA 02118, USA; apeksha.shenoy@bostonsp.com (A.S.); lily.arnett@bostonsp.com (L.A.); weiqi.jiao@bostonsp.com (W.J.); 4 PANTHERx Rare Pharmacy, Pittsburgh, PA 15275, USA; jguarinoni@pantherxrare.com; 5 Apellis Pharmaceuticals Inc., Waltham, MA 02451, USA; jinny.min@apellis.com; 6 Mayo Clinic, Rochester, MN 55905, USA; dingli.david@mayo.edu

**Keywords:** quality of life, healthcare resource utilization, hemoglobin, OPERA, FACIT, transfusions, PROMIS

## Abstract

**Background**: Paroxysmal nocturnal hemoglobinuria (PNH) is a rare, acquired, life-threatening disease characterized by complement-mediated hemolysis. OPERA is the first US longitudinal real-world study on C3 inhibitor therapy, known as pegcetacoplan. **Methods**: OPERA enrolled US patients with PNH, age ≥18, who were prescribed pegcetacoplan, and data were collected from routine care. Hemoglobin was reported by patients during regular follow-up (censored from transfusions). The Functional Assessment of Chronic Illness Therapy (FACIT)-Fatigue (0–52 score) and Patient-Reported Outcomes Measurement Information System scale for Cognitive Function Abilities (PROMIS-CF; 23.27–67.09 t-score) were completed electronically (low score = negative outcome). Patients self-reported incidence of healthcare resource utilization (HCRU). **Results**: By January 2024, 70 patients (mean age 44.6 years; 57.1% female) reported up to 9 months of pegcetacoplan treatment, with a median [IQR] follow-up of 6.6 [3.8] months. The latest reported hemoglobin levels improved by a mean (SD) of 2.6 (1.9) g/dL from baseline. At 3, 6 and 9 months, patients reported clinically meaningful improvements (≥5 points) in FACIT-F (53.3–69.0%) and (≥2 points) PROMIS-CF (46.7–55.2%). Patients reported a <10% incidence rate per person month of all HCRU events. **Conclusions**: This first longitudinal real-world US study indicates a positive trend in Hb, fatigue, and cognition with limited HCRU during pegcetacoplan treatment in adults with PNH.

## 1. Introduction

Paroxysmal nocturnal hemoglobinuria (PNH) is a rare, acquired, life-threatening disease characterized by complement-mediated intravascular and extravascular hemolysis and thrombosis. Many patients with PNH experience anemia as a result of chronic hemolysis, leading to persistent fatigue and a dependence on frequent blood transfusions [1,2]. These symptoms and experiences may negatively impact the overall quality of life (QOL) of patients. For instance, some individuals experience impaired cognitive function that may result in difficulties such as “brain fog” [3]. Furthermore, anemia-related PNH symptoms may require additional hospital and emergency room visits, ultimately leading to increased healthcare resource utilization (HCRU) [4].

Previous treatments for PNH include C5 inhibitor therapies such as eculizumab and ravulizumab; however, C5 inhibitors do not address extravascular hemolysis, resulting in persistent anemia in certain individuals [5,6]. The continued clinical manifestations of illness (e.g., fatigue, anemia, thrombotic events) and resulting strains on QOL in patients with PNH receiving C5 inhibitors demonstrates the need for more effective treatment [7]. More recently, pegcetacoplan (PEG) was the first approved C3 inhibitor therapy, acting at the level of C3, thereby reducing both intravascular and extravascular hemolysis [5,8]. Following clinical trial evidence demonstrating superior PNH symptom relief with PEG treatment in both treatment-naïve and C5-inhibitor-treated patients [5,9,10], PEG received approval in the United States by the Food and Drug Administration (FDA) as treatment for adults with PNH, as well as approval as a monotherapy by the European Medicines Agency for adults with PNH who have hemolytic anemia [8,11]. Open label clinical trials have also shown that PEG’s improved hematologic outcomes and favorable safety profiles extend over 48 weeks of treatment [12]. While clinical trials have assessed the efficacy of PEG, including the evaluation of patient-reported outcomes, the OPERA (Exploratory **O**utcomes Study in **P**aroxysmal Nocturnal Hemoglobinuria Using **E**MPAVELI^®^, a **R**eal-World **A**nalysis) study seeks to contribute to the limited available real-world PEG data.

This prospective observational study describes the first longitudinal real-world data on PEG treatment among adults with PNH in the United States. Given the value in recognizing patient experiences, and the propensity for PNH to substantially impact aspects of QOL, OPERA records PEG treatment over time through clinical values and patient-reported outcomes [3,13,14]. This nine-month analysis includes variables of treatment compliance, hemoglobin, fatigue, cognitive function, transfusion requirements, and HCRU in patients with PNH, over a period of up to nine months of PEG treatment, in the OPERA study.

## 2. Materials and Methods

### 2.1. Study Design and Objectives

**Figure 1:**
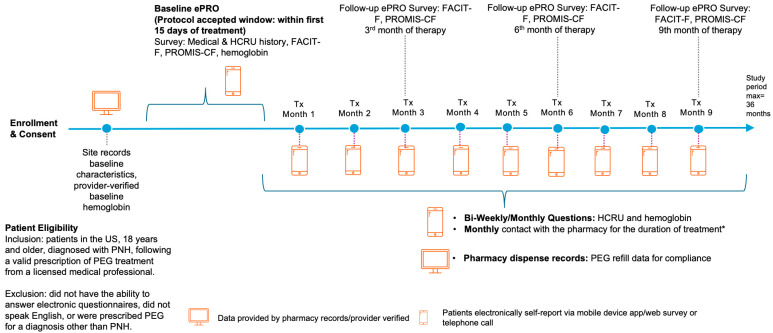
OPERA study design: patient selection and data collection. * Monthly contact between the patient and pharmacy was conducted regardless of study participation. Abbreviations: ePRO, electronic patient-reported outcomes; FACIT-F, Functional Assessment of Chronic Illness Therapy–Fatigue; HCRU, healthcare resource utilization; PNH, Paroxysmal Nocturnal Hemoglobinuria; PROMIS-CF, Patient-Reported Outcome Measurement Information System v2.0—Cognitive Function Abilities Subset 8a; Tx, Treatment; PEG, pegcetacoplan.

In accordance with guidelines from the International Society for Pharmacoeconomics and Outcomes Research (ISPOR) and International Society for Pharmacoepidemiology (ISPE) [15], as well as epidemiologic guidance from the FDA [16], our protocol, conduct, and analysis followed best practices for real-world observational exploratory pharmacoepidemiology studies. This included a priori protocol and analysis that comply with RECORD PE [17] and STROBE [18] reporting frameworks (Supplemental Table S1). OPERA is a nationally representative and centrally recruited opt-in study. Beginning in January 2022, eligible patients (Figure 1) who were prescribed PEG with referrals to a specialty pharmacy (PANTHERx Rare, Pittsburgh, PA, USA) were recruited to participate in the OPERA study. The PNH diagnosis was verified with the patient by the pharmacy, and patients were enrolled following electronic consent as approved by an institutional review board. This analysis includes data from patients who initiated treatment through 4 January 2024, completing ≥1 study activity, from baseline through up to 9 months of PEG treatment, though some patients may have had shorter available follow-up durations. OPERA patients received PEG in 1080 mg subcutaneous doses twice weekly or every 3 days. No formal sample size was required, as the objective of this study was to describe real-world treatment patterns, laboratory markers, and resource use utilizing descriptive statistics. Moreover, PNH is a rare disease, and a small sample was expected as investigators anticipated declines in longitudinal response rates [19].

### 2.2. Assessments and Data Collection

The OPERA study collected electronic patient-reported outcomes (ePROs) [20] at regular intervals (Figure 1), aligning with FDA standards [21]. Information was collected as part of routine care, and this study did not direct any medical interventions. Baseline ePRO data met protocol validity requirements for analysis if completed within ≤15 days of a patient’s first dispensing of PEG, to ensure that treatment effects did not confound initial assessments [9]. To assess an objective clinical marker of PNH, patients provided self-reported hemoglobin laboratory results, given previously demonstrated dependability [7]. Because this study collected data provided during routine care, hemoglobin results were obtained from patients only when available following laboratory testing performed at the discretion of a patient’s care provider. QOL outcomes related to fatigue and cognitive function were assessed using patient questionnaires. The Functional Assessment of Chronic Illness Therapy-Fatigue (FACIT-F) scale (0–52 total score) [22] was utilized given established use for PNH symptoms [5,7]. Exploratory cognitive function data were obtained through the use of the Patient-Reported Outcome Measurement Information System v2.0—Cognitive Function Abilities Subset 8a questionnaire (PROMIS-CF; 23.27–67.09 t-score) [23]. Scores for FACIT-F and PROMIS-CF were provided on a 5-point Likert scale, with lower scores representing greater levels of fatigue or cognitive impairment. PROMIS-CF total scores were standardized using a t-score metric according to the PROMIS-CF scoring manual. HCRU and the number of patients requiring transfusion were additional endpoints captured to establish the disease burden. Adverse events were captured during routine telephonic prescription refill calls or through spontaneous reporting by the patient to the specialty pharmacy. Patients were compensated according to fair market value [24] for their participation.

### 2.3. Data and Analysis

Descriptive statistics were used to analyze data from OPERA. This included means, standard deviations, medians, and interquartile ranges for continuous variables, while frequencies, counts, and incidence rates described categorical variables.

To establish treatment compliance, the days of medication supplied to the patient were divided by total days of availability, per pharmacy dispense records. The compliance period of interest began following two standard dispenses (28-day supplies) to account for initial logistical considerations, including onboarding/authorization delays, patient learning curve for administration, familiarity with injection timing, and the potential for patients who participated in clinical trials to utilize any remaining vials.

Hemoglobin data were censored in cases where patients reported receiving blood transfusions; only data prior to the first reported transfusion were eligible for inclusion. This approach is consistent with PNH clinical trial reporting, as transfusions are considered intercurrent events that may confound results by increasing hemoglobin amounts independent of treatment [5,25]. Patients providing both a baseline hemoglobin and ≥1 follow-up hemoglobin lab were included in the analysis. To assess normalization of hemoglobin values, the latest reported hemoglobin values were analyzed against gender-specific population norms (≥12 g/dL for females and ≥13 g/dL for males) [26].

Changes in reported QOL are considered clinically meaningful when scores improve by ≥5 FACIT-F total points [25,27,28] and ≥2 PROMIS-CF t-score points [29]. Therefore, the change from baseline per patient, between total points and t-score points at 3, 6 and 9 months, was assessed to identify clinically meaningful improvements in FACIT-F and PROMIS-CF, respectively. At each timepoint, individual scores were compared to the general population norms (FACIT-F, 39.5; PROMIS-CF, 50.0) to contextualize QOL outcomes. Responses to QOL measures were analyzed at the level of each item to determine the percent of patients reporting a ≥1-level improvement (on the Likert scale) at each follow-up [30], examining relevant symptom improvements associated with individual questions. Following pharmacoepidemiology best practices, HCRU data were also analyzed to capture incidence rates per person month with 95% confidence intervals [31], aligning with previous PNH burden analyses [4].

Survey responses and pharmacy call data were stored in a HIPAA-compliant, secure electronic data capture (EDC) system. Best practices for data quality control and completeness were conducted to ensure endpoint accuracy; therefore, data provided electronically, directly from patients (some fields requiring a response to minimize missing data), were screened for inaccuracies/outliers by qualified researchers in conjunction with site communications, as described previously [32,33]. Results may include patients who have chosen/been advised (by personal clinician) to discontinue participation. As a result of study data originating from routine care, “missing” data were expected, particularly given the variable frequency of laboratory testing between patients. All analyses reflect the qualifying data provided through routine communication with patients.

## 3. Results

### 3.1. Demographics

By 4 January 2024, 70 patients had enrolled in the OPERA study, completed up to 9 months of PEG treatment, and met activity requirements for inclusion in the overall analysis. The average age was 44.6 years, and 40 (57.1%) were female (Table 1). Overall, 65 (92.9%) patients were previously treated with C5 inhibitors. Prior to baseline (within 12 months), 39 (55.7%) patients required transfusions; among them, 79.5% (*n* = 31) reported spending ≥3 h at centers for transfusion care. The number of patients eligible for analysis with available data for the respective outcomes is further shown in Supplemental Figure S1.

### 3.2. Compliance

Assessing individual periods of interest, 62 patients completed sufficient refill requests to establish treatment compliance. Among them, the median (IQR) rate of PEG compliance was 100.0% (3.7%), demonstrated over 357.0 patient months.

### 3.3. Hemoglobin

**Figure 2:**
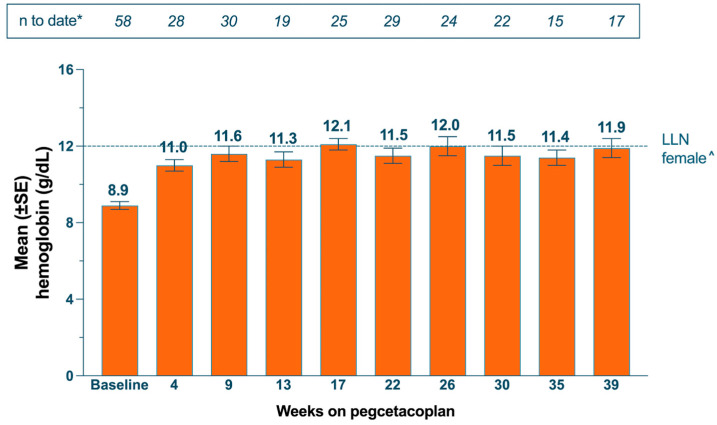
OPERA subgroup analysis of average hemoglobin levels reported by treatment duration. Data depicts summary statistics during routine care and monitoring, which varies as directed by physicians. Hemoglobin was reported if/when this lab test was available; data were censored for transfusion events (all values provided after the first-reported intercurrent event were excluded from outcomes). * *N* = individual hemoglobin laboratory results reported during each period of treatment. ^ Line represents the lower limit of normal for hemoglobin values found for females in the general population. Current range: 4–39 weeks (1–9 months) of pegcetacoplan treatment. Over this period, the mean (SD) change from baseline by the latest reported hemoglobin level was 2.6 (1.9) g/dL. Abbreviations: LLN, lower limit of normal; SE, standard error.

In this analysis, 58 patients met the hemoglobin reporting criteria, (Table 2) with a mean (SD) baseline hemoglobin level of 8.9 (1.8) g/dL; after a median (IQR) follow-up period of 6.6 (3.8) months, the mean (SD) latest reported hemoglobin level was 11.5 (2.2) g/dL, with a 2.6 (1.9) g/dL change from baseline. Among them, 35 (60.3%) patients reported a ≥2 g/dL increase from baseline by their latest-reported hemoglobin value. Including all available hemoglobin values (censored at the date of first-reported transfusions; *N* = 209) organized by PEG treatment duration (Figure 2), mean hemoglobin levels ranged from 11.0 to 12.1 g/dL through a period of up to 39 weeks. The results remained similar when patients reporting transfusions during PEG treatment were excluded from analysis altogether (Supplemental Table S2). There were no reports of breakthrough hemolysis in study participants.

### 3.4. Quality of Life Related to Fatigue and Cognitive Function

Of the total participants, 32 patients successfully completed FACIT-F and PROMIS-CF questionnaires, and 38 patients did not complete questionnaires within the eligible criteria window defined by the protocol (≤15 days of a patient’s first dispensing of PEG) or not at all. The mean (SD) baseline FACIT-F score among 32 patients completing their baseline ePRO per protocol timing was 28.4 (11.5) (Table 3). During their follow-up, the mean (SD) FACIT-F total score was 38.6 (10.9) at 3 months (*N* = 29), 36.3 (11.9) at 6 months (*N* = 20), and 34.9 (10.0) at 9 months (*N* = 15) of PEG treatment. The majority of responding patients reported a change from baseline of ≥5 points in FACIT-F score at all ePRO timepoints, with *n* = 20/29 (69.0%) at 3 months, *n* = 12/20 (60.0%) at 6, and *n* = 8/15 (53.3%) at 9 months of PEG treatment. After initiating treatment, 33.3%–58.6% of patients reported a FACIT-fatigue score ≥39.5. At each follow-up ePRO, ≥40% of patients reported a ≥1-level increase from baseline in 9 of the 13 FACIT-F items (Supplemental Table S3). Summary results for FACIT-F total scores, mentioned above, remained similar when all available responses, including protocol deviations (outside the protocol-defined baseline timeframe), were alternatively included in the analysis (Supplemental Table S4).

At baseline, 32 evaluable patients reported a mean (SD) PROMIS-CF t-score of 46.9 (10.8). During their follow-up, the mean (SD) PROMIS-CF t-score was 51.8 (10.0) at 3 months (*N* = 29), 49.8 (12.2) at 6 months (*N* = 20), and 48.3 (7.8) at 9 months (*N* = 15) of PEG treatment. At each ePRO timepoint, more than 40% of patients experienced a change of ≥2 points from baseline in PROMIS-CF t-score: *n* = 16/29 (55.2%) at 3 months, *n* = 10/20 (50.0%) at 6 months, and *n* = 7/15 (46.7%) at 9 months. Additionally, 40.0%–51.7% of patients reported a PROMIS cognitive t-score ≥50.0 after initiating treatment. At all follow-up ePROs (3, 6, and 9 months), ≥40% of patients reported a ≥1-level increase from baseline in response to “My mind has been as sharp as usual”, “My memory has been as good as usual”, and “My thinking has been as fast as usual” (Supplemental Table S5). PROMIS-CF t-score summary findings were also mirrored when all available responses, including protocol deviations, were alternatively included for analysis (Supplemental Table S4).

### 3.5. Transfusion Requirements and Healthcare Resource Utilization

At baseline, 39 (55.7%) patients reported requiring transfusions prior to the study (Table 4). At the time of analysis, 69 OPERA patients provided follow-up HCRU data, with only 14 (20.3%) requiring transfusions (over varied treatment durations). Furthermore, over 556.4 patient months, OPERA patients reported low incidence rates per person month of transfusions (0.09), ER visits (0.03), and hospitalizations (0.02) while on PEG treatment.

## 4. Discussion

OPERA is the first study to collect longitudinal real-world data on patients with PNH in the United States currently undergoing treatment with the C3 inhibitor therapy, PEG. The overwhelming majority of patients reported previous C5 inhibitor treatment, indicating that these patients were not treatment-naïve prior to enrollment in the current study. Accompanying data in these individuals suggest that prior treatments failed to alleviate PNH symptoms and healthcare burdens, as demonstrated by the prevalence of anemia, diminished QOL, and need for transfusions prior to PEG therapy. After up to nine months of highly compliant PEG treatment, patients reported hemoglobin improvement, with many patients achieving normalization of hemoglobin levels. OPERA patients also reported improvement in aspects of QOL, including fatigue and cognitive function outcomes, freedom from transfusions, and limited HCRU. This analysis provides the basis for a comprehensive understanding of the longer-term results of PEG treatment for PNH, for the benefit of patients and providers.

Using hemoglobin response as an indicator of hemolysis and bone marrow failure, which are key manifestations of disease progression [35], OPERA patients experienced clinical improvements during PEG therapy. Hemoglobin improvement was sustained up to month 9 of PEG therapy, as demonstrated by the majority of patients’ latest reported laboratory values. These improvements indicate that continued, compliant therapy prolonged positive outcomes in patients. Notably, nearly half of patients (48.3%) reported their latest hemoglobin levels demonstrating normalization (by gender-specific standards), suggesting that many patients achieved non-anemic hemoglobin levels during PEG treatment. When reported hemoglobin values were evaluated by treatment duration, average levels remained consistent and improved throughout the study period (as early as month 1, through month 9). The current findings on hemoglobin improvements are consistent with previous studies [10], including head-to-head comparison versus C5i [5], and long-term data that showed improvement in mean hemoglobin of >2 g/dL following up to 3 years of treatment [36].

Notably, breakthrough hemolysis is an important consideration for treatment with compliment targeted therapies, which was first observed with C5 inhibitors [37,38]. Prior findings from the PEGASUS trial showed that 19 out of 80 patients treated with PEG experienced hemolysis-adverse events during either the randomized controlled or open-label periods; many cases were moderate in severity, while discontinuation occurred due to hemolysis in 5 patients [12]. In real-world patients receiving PEG as second-line therapy due to anemia experienced during C5 inhibition, one study revealed that 13 out of 48 patients experienced breakthrough hemolysis while receiving PEG or combination therapy with C5 inhibitors [39]. Notably, these findings from real-world practice in patients receiving PEG as second-line therapy included patients who were ineligible for clinical trials [39]. The current findings did not reveal any cases of breakthrough hemolysis among OPERA study participants. A study assessing treatment breakthrough hemolysis showed that dose escalation of PEG was an effective management strategy, whereby intensive PEG therapy resolved all adverse events associated with the breakthrough event [38]. Collectively, additional research is still needed to determine the incidence of breakthrough hemolysis in real-world practice and best management practices during PEG treatment.

Overall, many patients experienced improvements in self-reported measures of fatigue and cognitive function. In prior analyses of PEGASUS, after 16 weeks, patients treated with PEG reported greater improvement in FACIT-F item-level and total scores than eculizumab-treated patients [25,27]. OPERA patients exhibited similar clinically meaningful responses [25,27], with the majority reporting total scores improving by ≥5 points from baseline at each follow-up survey, demonstrating sustained meaningful changes in fatigue throughout long-term, real-world PEG treatment. At each follow-up ePRO, ≥30% of OPERA patients achieved FACIT-F total scores equivalent to the general population (39.5) [40], indicating an ability to improve fatigue symptoms with PEG therapy, minimizing an impairing symptom of PNH. Moreover, given the potential for response shifts/ceiling effects, whereby improved symptoms may have adjusted patients’ frame of reference over time [34,41], third-month QOL results may be the most indicative of our study population’s experience. Previous work has also highlighted the impaired cognitive function associated with PNH [7,40]. In order to analyze the previously reported symptoms of “brain fog” more closely [3], OPERA was the first longitudinal PNH study in the United States to collect ePROs using the PROMIS-CF measure. Of note, 46.7%- 55.2% of OPERA patients exhibited a clinically meaningful difference of ≥2 t-score points [29] at each follow-up ePRO, compared to baseline. After initiating PEG therapy, 40.0%–51.7% of patients met or exceeded the general population’s mean PROMIS-CF t-score of 50.0 [40], demonstrating improved/standard cognitive functioning. The QOL results seen in OPERA may have further implications, as PEG treatment has been associated with additional quality-adjusted life years compared to eculizumab and ravulizumab treatment [42]. This is accompanied by corresponding lifetime cost-savings, exemplifying additional potential long-term benefits for patients with PNH on PEG [42].

Transfusion dependence can impose restrictions on a patient’s life, and the limited treatment response to C5 inhibitor therapies has proven costly to patients [43,44]. Previous investigations found that 52.9% of patients with PNH treated with eculizumab and 32.7% of ravulizumab-treated patients still required ≥1 transfusions [43]. In contrast, the majority (79.7%) of OPERA patients were transfusion-free during 556.4 person months of PEG treatment. Minimal transfusion incidence was expected given similar findings noted in previous long-term PEG data [36,45]. Considering the time spent at transfusion centers prior to enrollment (79.5% among those requiring transfusion reported spending ≥3 h), transfusion freedom further decreases the inconveniences patients face. Furthermore, the incidence of transfusions, ER visits, and hospitalizations all contribute to the growing burden for patients and healthcare systems. When assessing the real-world costs for eculizumab- and ravulizumab-treated patients with PNH, combined care and drug costs amounted to USD 711,785 and USD 624,911, annually (respectively) [43]; moreover, shifting from eculizumab to PEG proved cost-effective, allowing patients exhibiting a suboptimal treatment response to advance to a good-to-complete response [44]. In OPERA, patients experienced less than 10% incidence rates per person month of transfusions, all-cause ER visits, and all-cause hospitalizations. Although the current study only had up to 9 months of follow-up data available, extrapolation of our findings would still indicate lower HCRU with PEG compared to prior findings in patients receiving eculizumab or ravulizumab treatment, with ~21% to 38% experiencing hospitalization and ED visits during a 12-month follow-up period [43]. The long-term HCRU results seen in OPERA thus far suggest that real-world patients on PEG may not only benefit from well-documented economic savings, but also the enhanced aspects of QOL and freedom associated with higher treatment response and minimal added care.

### Limitations

This analysis has several limitations. Due to the rare nature of PNH, and a longitudinal study design (such that OPERA is both ongoing and currently enrolling new patients), sample sizes varied by treatment duration and declined in later follow-up periods. Moreover, sample sizes were also impacted by limitations inherent to self-reported outcomes research (e.g., item nonresponse and entry errors) and eligibility criteria for consideration in analysis (e.g., hemoglobin values following blood transfusion); in some cases, this prevented the analysis of every response event from each patient. For instance, the current approach did not allow for collection of hemoglobin laboratory values at all timepoints, as these were only reported by participants following testing that was conducted at the discretion of their care providers. The findings were also subject to bias inherent to patient-reported outcomes (e.g., selection or non-response bias). Convenience sampling was utilized in accordance with the prescription process, wherein treating physicians determined patient eligibility to receive PEG, thus limiting generalizability. The ability to capture adverse events was limited to routine follow-up prescription refill calls and spontaneous reporting by the patient, which may have limited insights into the occurrence of potential adverse events. Also, not all aspects of medical care were able to be captured (e.g., transfusion units); future studies capable of linking patient health records with ePROs would provide useful insights into the impact of treatment on patient well-being. To address some of the potential limitations inherent to ePRO research, our approach involved multiple efforts to maintain patient contact consistently over the study period, minimize patient responsibility and recall, and to provide continuous real-time opportunities for reporting. This included regular outreach from the site and automatic triggering of ePRO survey questions (with response windows, tiered logic, and reminders) [46]. Notably, ePROs are a supported methodology [47,48] that limits investigational interference and provides real-world longitudinal evidence. Ultimately, the reporting rates for electronic-only, patient-reported measures in studies of similar designs legitimize trends seen in OPERA, particularly for a longitudinal study [19]. Collectively, these findings provide integral information on the patient experience, using measures including FACIT-F with previously demonstrated validity and reliability [13,49].

## 5. Conclusions

The OPERA study describes longitudinal real-world treatment patterns, laboratory markers, and resource use in US patients with PNH undergoing PEG therapy. Over a period of up to nine months of PEG therapy, patients reported improvements in hemoglobin, with many achieving normalization. OPERA provides real-world evidence that patients receiving long-term PEG therapy report clinically meaningful improvements in fatigue (FACIT-F) and cognitive function (PROMIS-CF). Patients also self-reported low incidence rates per person month of transfusions and other HCRU, indicating positive treatment responses to PEG therapy. In real-world patients treated outside of the rigors of a clinical trial protocol, these findings demonstrate that PEG continues to exhibit long-term positive effects on PNH outcomes. The literature would benefit from future real-world studies collecting PEG data for longer treatment durations.

## Figures and Tables

**Table 1:** Demographics of OPERA patients.

Baseline Characteristics	Total *N* = 70
**Age, years**	
Mean (SD)	44.6 (16.1)
**Gender, *n* (%)**	
Female	40 (57.1)
Male	30 (42.9)
**Previous complement inhibitor treatment, *n* (%)**	
Eculizumab	43 (61.4)
Ravulizumab	14 (20.0)
Eculizumab and Ravulizumab	8 (11.4)
None	5 (7.1)

Abbreviations: SD, standard deviation.

**Table 2:** OPERA hemoglobin levels ^1^.

	Baseline	Latest Reported Values ^2^
**Hemoglobin, g/dL**	*N* = 58 ^3^	*N* = 58 ^4^
Mean (SD)	8.9 (1.8)	11.5 (2.2)
Median	8.7	12.1
**Hemoglobin change from baseline**		
Mean (SD), g/dL	-	2.6 (1.9)
Patients with ≥1 g/dL improvement, *n* (%)	-	47 (81.0)
Patients with ≥2 g/dL improvement, *n* (%)	-	35 (60.3)
**Hemoglobin normalization, *n* (%)**		
Study population ^5^	2 (3.4)	28 (48.3)
**Follow-up period up to latest reported** **hemoglobin value, months**		
Median (IQR)	-	6.6 (3.8)

^1^ Patients met the criteria for analysis if they reported a baseline and ≥1 follow-up hemoglobin value. Hemoglobin is reported if/when this lab test is available, which varies (physician-directed) during routine care and monitoring. ^2^ After initiating pegcetacoplan treatment, censored for transfusion events, all values provided after the first-reported intercurrent event were excluded from these outcomes/hemoglobin analysis. ^3^ At baseline, 34 patients previously required transfusions. ^4^ After initiating PEG, 13 patients’ data were censored due to intercurrent events (transfusions). ^5^ Patients at or above the lower limit of normal hemoglobin found in the general population; 12 g/dL for females and 13 g/dL for males [26]. Abbreviations: IQR, interquartile range; SD, standard deviation.

**Table 3:** OPERA patient quality-of-life outcomes in the per-protocol population, by duration of pegcetacoplan treatment ^1^.

		Baseline ^2^ *N* = 32	3 Months *N* = 29	6 Months *N* = 20	9 Months *N* = 15
**FACIT-Fatigue** (*N* = 32 ^3^)	**Total score *n* (%)**				
	Mean (SD)	28.4 (11.5)	38.6 (10.9)	36.3 (11.9)	34.9 (10.0)
	Median	27	40	38	37
	**Change from baseline, *n* (%)**				
	≥5 points	-	20 (69.0)	12 (60.0)	8 (53.3)
	**General population comparison, *n* (%)**				
	Total score ≥39.5 points	6 (21.4)	17 (58.6)	10 (50.0)	5 (33.3)
**PROMIS-CF** (*N* = 32 ^3^)	**T-score**				
	Mean (SD)	46.9 (10.8)	51.8 (10.0)	49.8 (12.2)	48.3 (7.8)
	Median	44.8	50.2	48.2	48.2
	**Change from baseline, *n* (%)**				
	≥2 points	-	16 (55.2)	10 (50.0)	7 (46.7)
	**General population comparison, *n* (%)**				
	Total score ≥50.0 points	10 (31.3)	15 (51.7)	9 (45.0)	6 (40.0)

^1^ Analysis did not adjust for response shift or ceiling effects in quality-of-life outcomes [34]. ^2^ Per-protocol, a valid baseline ePRO, was completed in ≤15 days from the first pegcetacoplan dispense date. ^3^ Minor differences were seen in patient demographics between those providing protocol-compliant baseline ePROs and those that did not (age was similar but m:f ratio was 50:50 for patients that did not provide a valid baseline). Abbreviations: FACIT-F, Functional Assessment of Chronic Illness Therapy–Fatigue; PROMIS-CF, Patient-Reported Outcome Measurement Information System v2.0—Cognitive Function Abilities Subset 8a; SD, standard deviation.

**Table 4:** Transfusion requirements and healthcare resource utilization for OPERA patients.

	Value
**Patients requiring transfusions, *n* (%)**	
Baseline ^1^ (*N* = 70)	39 (55.7)
During PEG therapy, follow-up (*N* = 69 ^2^)	14 (20.3)
**Healthcare resource utilization reported *during PEG therapy***	
**Transfusions**	
Patients reporting event, *n* (%)	14 (20.3)
IR ^3^ (95% CI)	0.09 (0.07–0.12)
**ER visits**	
Patients reporting event, *n* (%)	11 (15.9)
IR ^3^ (95% CI)	0.03 (0.02–0.04)
**Hospitalizations**	
Patients reporting event, *n* (%)	7 (10.1)
IR ^3^ (95% CI)	0.02 (0.01–0.03)
**Duration of PEG therapy/follow-up**	
Person months	556.4

^1^ Recall window = up to 12 months prior. ^2^ One patient did not have follow-up HCRU data available at the time of analysis. ^3^ Per person month. Abbreviations: CI, confidence interval; ER; emergency room; IR: incidence rate; PEG, pegcetacoplan.

## Data Availability

The data that support the findings of this study are available on request from the corresponding author. The data are not publicly available due to privacy or ethical restrictions.

## Supplementary Materials

The following supporting information can be downloaded at: https://www.mdpi.com/article/10.3390/hematolrep16040065/s1, Table S1: The RECORD statement for pharmacoepidemiology (RECORD-PE) checklist; Table S2: OPERA hemoglobin levels (cross-sectional analysis; patients reporting any transfusions excluded); Table S3: OPERA: FACIT-F item-level analysis in the per-protocol population; Table S4: OPERA patient follow-up quality-of-life outcomes including protocol deviations; Table S5: OPERA: PROMIS-CF item-level analysis in the per-protocol population; Figure S1: Flow chart of number of patients at enrollment to patients eligible for analysis with available data for the respective study outcomes.
